# Innovative prolonged-release oral alkalising formulation allowing sustained urine pH increase with twice daily administration: randomised trial in healthy adults

**DOI:** 10.1038/s41598-020-70549-2

**Published:** 2020-08-18

**Authors:** C. Guittet, C. Roussel-Maupetit, M. A. Manso-Silván, F. Guillaumin, F. Vandenhende, L. A. Granier

**Affiliations:** 1 Advicenne, Nîmes, France; 2 ClinBay, Genappe, Belgium

**Keywords:** Drug discovery, Nephrology, Urology

## Abstract

A multi-particulate fixed-dose combination product, consisting of a combination of two alkalising salts formulated as prolonged-release granules, ADV7103, was developed to obtain a sustained and prolonged alkalising effect. The specific release of both types of granules was shown in vitro through their dissolution profiles, which indicated that potassium citrate was released within the first 2–3 h and potassium bicarbonate up to 10–12 h after administration. The long-lasting coverage of ADV7103 was confirmed through a randomised, placebo-controlled, double-blind, two-period study, measuring its effect on urine pH in healthy adults (n = 16) at doses of alkalising agent ranging between 0.98 and 2.88 meq/kg/day. A significant increase of urine pH with a positive dose–response in healthy adult subjects was shown. Urine pH above 7 was maintained during 24 h with a dosing equivalent to 1.44 meq/kg twice a day, while urine pH was below 6 most of the time with placebo. The effect observed was non-saturating within the range of doses evaluated and the formulation presented a good safety profile. ADV7103 provided an effective prolonged release of alkalising salts to cover a 12-h effect with adequate tolerability and could afford a twice a day (morning and evening) dosing in patients requiring long-term treatment.

## Introduction

Current alkalising treatments in adults and children generally consist of immediate-release forms of citrate or bicarbonate salts. The absorption of the actives is rapid, generating a peak of high alkaline load, and a short-lived effect. These treatments are characterized by inconvenient dosing schemes (3–6 daily doses), difficult day and night coverage and a jerky action leading to a variable efficacy, gastro-intestinal discomfort, and bad taste, which may result in poor acceptability and compliance^1,2^.

After oral administration, citrate undergoes oxidative metabolic breakdown to carbon dioxide (CO_2_) or bicarbonate. Consequently, a basifying effect is associated with its metabolism^3^. This salt is mainly absorbed under its divalent form (pH 4.8–6.4), and is thus known to have an absorption window limited to the upper side of the small intestine (duodenum, early part of the jejunum)^4^. At physiological blood pH (7.4), citrate is entirely ionized in its trivalent form. Most of the citrate in the blood circulates unbound at relatively low (0.05 to 0.3 mmol/l) concentrations and the remaining quota is bound to calcium, potassium and sodium^5^.

In contrast, oral bicarbonate is absorbed all over the gastro-intestinal tract, independently of the local pH. A massive elimination of bicarbonate could occur in the stomach, since it neutralizes gastric acid with the production of CO_2_ eliminated by the respiratory route. The remaining bicarbonate not involved in that reaction is rapidly absorbed by the intestinal mucosa^6–8^.

In order to maintain a sustained alkalising effect over 12 h with a good safety profile, ADV7103, a new prolonged-release oral formulation, was designed to maximise absorption of the active substances. ADV7103 formulation consists of potassium citrate (CK) prolonged-release granules and potassium bicarbonate (BK) prolonged-release granules. After administration of the drug product, the active substances are released with an optimised release profile for each substance throughout the gastro-intestinal tract.

The objective of this work was to demonstrate the prolonged-release action of ADV7103 through its in vitro dissolution profile and the long-lasting alkalising effect of ADV7103 administered twice daily in healthy adults by evaluating the pharmacodynamic effect on urine pH values.

## Methods

### Formulation development

A new oral formulation, combining the advantages of potassium citrate and potassium bicarbonate was developed. Two types of prolonged-release granules (in practice 2-mm diameter tablets) were developed separately, each containing one active substance: green granules containing approximately 67% of potassium citrate (tri-potassium 2-hydroxypropane-1,2,3-tricarboxylate monohydrate) and white granules containing approximately 66% of potassium bicarbonate (potassium hydrogen carbonate). Two different colours were used in order to distinguish both types of granules, mainly for quality control purposes.

Prolonged release was achieved by coating the granule core with a polymer film offering a versatile diffusion barrier for both drugs. A pH-independent water-insoluble ethylcellulose film layer was used as main limiting factor of the diffusion of the highly soluble drug substances by virtue of its semi-permeable nature.

As the dissolution rate is mainly a function of the thickness of the coating film, the amount of ethylcellulose was defined for each type of granules to achieve the appropriate dissolution profile and to maximise overall drug absorption. The respective target dissolution profiles were conceived according to the physiological characteristics of their active ingredients in order to avoid an absorption peak and obtain continuous efficacy of the combination during 12 h, in order to allow a twice-a-day administration, while limiting the burst effect.

Potassium citrate prolonged-release granules were developed to release potassium citrate within 3 h, to be absorbed mainly in the upper part of the small intestine. They were obtained by direct compression involving microcrystalline cellulose as binder, together with lubricants and flowing agents and then coated. The release mechanism consists in solubilisation of potassium citrate after diffusion of water through the semi-permeable film layer and then diffusion of the active through the polymer at a slow rate.

Potassium bicarbonate prolonged-release granules were developed to release potassium bicarbonate within 12 h. Due to the particularly high solubility of potassium bicarbonate, the release mechanism of potassium bicarbonate was based on a combination of the properties of an hypromellose sustained-release matrix and of the ethylcellulose coating. Upon diffusion of water through the semi-permeable ethylcellulose film layer, the hydrophilic hypromellose matrix hydrates on its outer surface to form consecutively degraded gel layers, thus controlling the influx of additional water. The semi-permeable film layer, in turn, controls the diffusion of the active out of the granules. Potassium bicarbonate granules were obtained by direct compression involving the hypromellose matrix, microcrystalline cellulose as binder, together with lubricants and flowing agents and subsequently coated.

In both cases, the coating solution, consisting of ethylcellulose 5% in ethanol, was directly sprayed onto the granules under rotation using a coating pan with a spraying system under predefined and controlled process parameters.

The final formulation was obtained as a fixed-dose combination of the two types of granules, containing roughly one part of potassium citrate and two parts of potassium bicarbonate.

### In vitro dissolution testing

In order to assess the dissolution profile of each type of granules, in vitro dissolution methods were developed on the basis of dissolution tests for prolonged-release solid dosage forms described in the European Pharmacopoeia. Sink conditions were verified. Approximately 350 granules were placed in each vessel of a USP type 2 rotating paddle dissolution apparatus for a volume of 1 l of purified water. They were stirred at 100 rpm, 37 °C (± 0.5 °C). Samples were drawn after 15 min, 30 min, 1 h, and 2 h for potassium citrate, and after 1 h, 5 h, and 10 h for potassium bicarbonate. In both cases the amount of potassium released was measured. For potassium citrate, potassium release was determined after dilution of the samples with purified water using an XP flame photometer with a potassium filter (BWB Technologies Ltd, Newbury, UK). For potassium bicarbonate, determination of potassium release was performed by conductivity using an Orion Star Plus meter (Thermo Fisher Scientific, Beverly, MA, USA).

### Phase I study in healthy adults

A randomised, placebo-controlled, double-blind, two-period study with an adaptive design, was conducted to investigate the pharmacodynamics of the product and its safety and tolerability in healthy adult subjects (trial registration on the 18/09/11 as EudraCT 2011-004679-35). The study was approved by the French South-East regional independent ethics committee and regulatory authorities, and conducted in accordance with Good Clinical Practice and the Declaration of Helsinki. Signed written informed consent was obtained from all subjects. The study population consisted of 16 healthy adult subjects, male or female, aged 18–55 years, with a BMI of 18–30 kg/m^2^, without major medical history or chronic treatment.

The primary objective was to assess the pharmacodynamic effect on urine pH (measure using a validated pH-metre) of six oral doses or dose regimens of ADV7103 versus placebo after 4–5 days of treatment. The secondary objectives were to assess the dose–response relationship of ADV7103 on urine pH after 4–5 days of treatment, to assess the pharmacodynamic effects of oral doses of ADV7103 on other urine parameters after 4–5 days of treatment, to assess the residual effect of oral doses of ADV7103 on urine pH after treatment discontinuation over a 24-h period, and to assess safety and tolerability of oral doses of ADV7103. All data were collected by qualified personnel at Eurofins Optimed facility.

Doses of ADV7103 were pre-specified for Period I, and doses and/or dose regimens of ADV7103 for Period II were determined further to interim review of safety and pharmacodynamic (PD) data, in order to assess any potentially improved dose or dose regimen.

**Figure 1:**
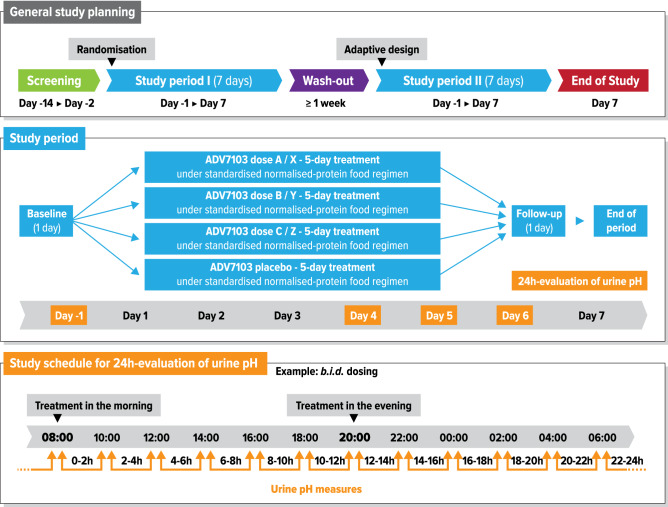
Design of B03CS study. Doses of CK/BK in Period I (administered morning and evening in all cases): (A) 17/34 mg/kg b.i.d (0.98 meq/kg/day), (B) 33/66 mg/kg b.i.d. (1.90 meq/kg/day), (C) 50/100 mg/kg b.i.d. (2.88 meq/kg/day). Doses of CK/BK and dose regimens in Period II (2.38 meq/kg/day in all cases): (X) 41.5/83 mg/kg b.i.d. morning and evening, (Y) 41.5/83 mg/kg b.i.d. midday and bedtime, (Z) 33/66 mg/kg in the morning and 50/100 mg/kg in the evening.

Each of the two study periods included a day to assess baseline effects (Day-1) followed by 5 days of treatment (Day 1 to 5). Reversibility was assessed during 24-h after the end of each treatment period (Day 6). A wash-out period of at least one week was planned between the two study periods. The end of study visit was planned on the last day of Period II (Day 7) (Fig. 1).

During each study period, urine pH was measured every 2 h for 24 h: at baseline (before treatment, Day – 1), during the two last treatment days (Day 4 and Day 5), and during follow-up after treatment was stopped (Day 6). Subjects were randomized in a balanced manner among four selected treatment sequences. All treatments were administered under a standardised protein-normalised food regimen.

Three ADV7103 dosing arms were tested compared to placebo during Period I, all administered morning and evening: a low dose consisting of 17 mg/kg CK and 34 mg/kg BK *b.i.d.* (corresponding to a total of 0.98 meq/kg/day); an intermediate dose of 33 mg/kg CK and 66 mg/kg BK *b.i.d.* (1.90 meq/kg/day); and a high dose of 50 mg/kg CK and 100 mg/kg BK *b.i.d.* (2.88 meq/kg/day).

An additional ADV7103 dose level between the intermediate and the high dose (corresponding to 2.38 meq/kg/day), was tested during Period II versus placebo under three different dosing regimens: a first dosing regimen with two equal doses of 41.5 mg/kg CK and 83 mg/kg BK *b.i.d.,* morning and evening; a second dosing regimen with the same doses 41.5 mg/kg CK and 83 mg/kg BK *b.i.d.* delayed by approximately 2 h, midday and bedtime; and a third dosing regimen with two different doses, 33 mg/kg CK and 66 mg/kg BK in the morning and 50 mg/kg CK and 100 mg/kg BK in the evening.

Both subjects and care providers in charge of the assessments were blinded. Blinding was ensured, when required, by the addition of a dose of placebo to the assigned doses.

Urine citrate, calcium, phosphate, potassium and sodium were measured on 24 h-urine collections and were compared to baseline values. The incidence of urine pH > 8.0 was evaluated. Vital signs and ECG parameters were recorded. Adverse events were reported, with particular focus on gastro-intestinal tolerability.

### Statistical methods

The PD analysis set included all subjects who received treatment and had valid PD data. It was estimated that 4 subjects per treatment were required to provide approximately 83% power to detect a mean urine pH difference of 1 between any ADV7103 dose group and placebo at the two-sided 5% significance level at the end of Period I. Powering assumed a residual standard deviation equal to 0.4, based on literature data^9,10^, and no correction for multiple comparisons.

Randomisation to one of the four possible treatment sequences (see Table 1) was performed in a balanced manner and in accordance with a randomisation table. Subjects of both genders were enrolled, including at least 40% of male and 40% of female subjects, with stratification by gender, so that at least a female and a male subject are assigned to each treatment sequence. The randomisation table was supplied in a sealed envelope by the study statistician to the pharmacist in charge of the preparations.

**Table 1:** Demographic characteristics for each sequence group.

Sequence Period I Period II	Sequence 1 placebo Regimen Y	Sequence 2 dose A Regimen X	Sequence 3 dose B Placebo	Sequence 4 dose C Regimen Z	Total
n	4	4	4	4	16
**Age (years)**					
Mean	27.5	31.8	30.5	39.8	32.4
SD	7.23	10.8	10.4	13.8	10.8
Minimum	19	26	24	23	19
Median	27.5	26.5	26.0	41.5	27.0
Maximum	36	48	46	53	53
**Sex**					
Female	2 (50.0%)	1 (25.0%)	2 (50.0%)	2 (50.0%)	7 (43.8%)
Male	2 (50.0%)	3 (75.0%)	2 (50.0%)	2 (50.0%)	9 (56.3%)
**Ethnic origin**					
Caucasian	4 (100.0%)	4 (100.0%)	4 (100.0%)	4 (100.0%)	16 (100.0%)
**Height (cm)**					
n	4	4	4	4	16
Mean	171.5	173.0	169.8	173.3	171.9
SD	9.2	4.2	10.1	6.7	7.2
Minimum	159	168	158	167	158
Median	173.0	173.5	170.5	173.0	173.0
Maximum	181	177	180	180	181
**Weight (kg)**					
n	4	4	4	4	16
Mean	71.4	64.9	68.1	62.1	66.6
SD	6.5	4.6	4.1	6.7	6.1
Minimum	66.2	58.7	64.3	54.9	54.9
Median	69.2	65.7	67.2	61.9	66.6
Maximum	80.9	69.6	73.6	69.8	80.9
**BMI (kg/m**^**2**^**)**					
n	4	4	4	4	16
Mean	24.3	21.8	23.8	20.7	22.6
SD	1.5	2.5	2.9	2.2	2.6
Minimum	22.8	18.7	19.8	18.5	18.5
Median	24.1	22.3	24.5	20.5	23.4
Maximum	26.2	23.8	26.4	23.4	26.4

Doses of CK/BK in Period I (administered morning and evening in all cases): (A) 17/34 mg/kg b.i.d (0.98 meq/kg/day), (B) 33/66 mg/kg b.i.d. (1.90 meq/kg/day), (C) 50/100 mg/kg b.i.d. (2.88 meq/kg/day). Doses of CK/BK and dose regimens in Period II (2.38 meq/kg/day in all cases): (X) 41.5/83 mg/kg b.i.d. morning and evening, (Y) 41.5/83 mg/kg b.i.d. midday and bedtime, (Z) 33/66 mg/kg in the morning and 50/100 mg/kg in the evening.

The mean pH measures of the 2 h urine collections were plotted in order to represent the evolution of pH during 24 h on Day 4 (during treatment), and Day 6 (return to baseline).

The four co-primary PD endpoints were subsequently calculated from the urine pH measures performed every 2 h during 24 h on Day 4 and Day 5 separately and then pooled. These four co-primary endpoints were: (i) the mean 24-h urine pH values, (ii) the urine pH values in the first morning urines, (iii) the percentage of urine samples over a 24-h period for which the observed pH was ≥ 7.0, and (iv) the urine pH fluctuation over a 24-h period (calculated as the difference between the maximum and the minimum pH values in that interval). Baseline values were similarly derived for the urine parameters on Day − 1 of each study period. Return to baseline urine pH (as determined by the mean pH values on Day 6) and mean change from baseline in citraturia values were secondary endpoints.

Three of the co-primary endpoints, the 24-h mean, first morning values, and the fluctuation of urine pH, as well as the secondary endpoint change in citraturia, were compared between treatments through a mixed-effect analysis of covariance (ANCOVA) model, including the baseline as covariate, the treatment, the study period and the sequence as fixed effects and the subject as random effect. Least square means were reported by treatment with 95% confidence intervals. Treatment differences compared to placebo were reported with 95% confidence intervals and the significance of the difference was assessed at the 2-sided 5% level.

Dose–response analyses for the three co-primary urine pH endpoints indicated above were performed using a mixed effect E_max_ model fitted to the data with the subject as random effect, according to the formula:

$${E}_{ij}=E0+Emax*\frac{dose}{ED50+dose}+{r}_{i}+{\varepsilon }_{ij}$$

where ${E}_{ij}$ is the value for subject *i* at period *j,* $E0$ is the placebo response, $Emax$ is the maximum change versus placebo, $ED50$ is the dose producing 50% of the maximum change, ${r}_{i}$*is the subject level random effect, and *${\varepsilon }_{ij}$* is the residual.*

Parameter estimates and predictions were reported with 95% confidence intervals. The doses required to obtain mean pH values of 7.0 or 7.5 and doses affording a pH increase of 0.2 or 0.5 units were derived from the models through inverse prediction.

The proportion of urine pH values ≥ 7.0 was calculated and analysed with a mixed-effect logistic regression model for binomial endpoints. Two models were applied: one with the treatment as categorical factor and one with the dose as a covariate, both with a random subject effect.

Safety data were summarised using standard descriptive methods. All statistical analyses were generated using SAS software, Version 9 for the SAS System for Windows (SAS is a registered trademark of SAS Institute Inc., Cary, NC, USA).

## Results

A fixed-dose combination was developed, containing potassium citrate and potassium bicarbonate, to be released throughout the gastro-intestinal tract during 12 h.

### Dissolution profiles

**Figure 2:**
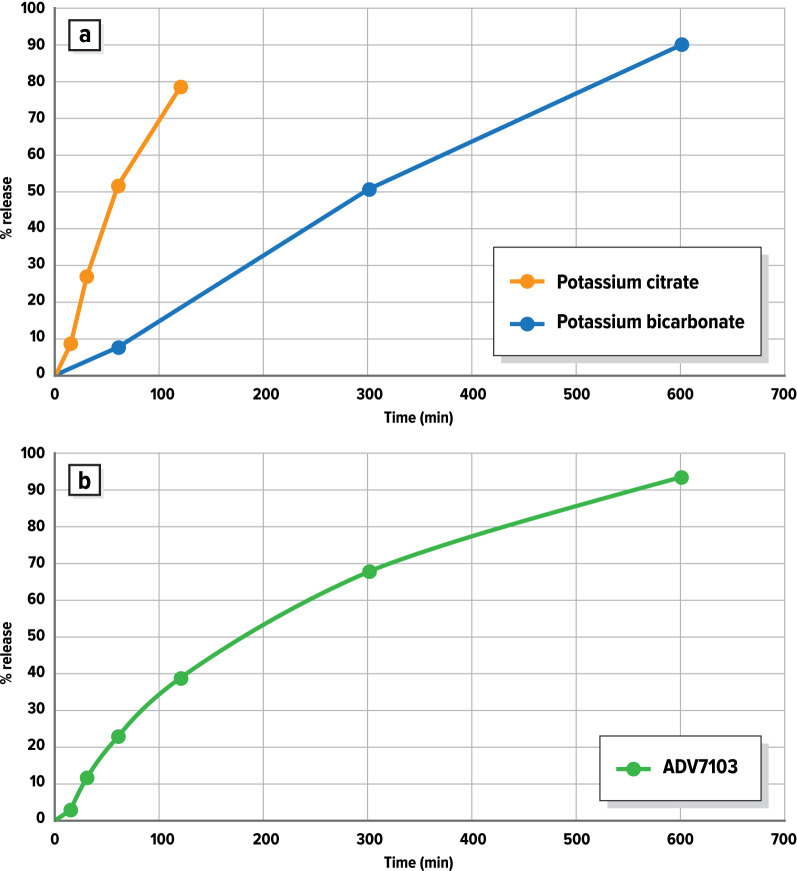
In vitro dissolution profiles of: (**a**) batches of prolonged-release granules of potassium citrate and potassium bicarbonate used in ADV7103 clinical batch; (**b**) ADV7103 formulation (calculated by combining both release profiles in adequate proportions).

The in vitro dissolution profile of potassium citrate granules indicated that the release of potassium citrate was lengthened to 2–3 h (79% release after 120 min, Fig. 2a) to target absorption of the drug in the upper intestine. The release of potassium bicarbonate according to its in vitro dissolution profile was limited during the first h (8% release at 60 min) to be prolonged over at least 10 h (90% release after 600 min, Fig. 2a), in order to achieve the long duration of action needed to cover a twice-a-day administration. The release profile of ADV7103 confirmed the prolonged-release of the combined formulation (94% release after 600 min, Fig. 2b).

### Phase I study population

The formulation was tested in adult subjects in order to confirm the adequate alkalising treatment during 12 h. A total of 42 subjects were screened, from which 16 subjects (9 males and 7 females), aged from 19 to 53 years, all complying with inclusion criteria, were randomised (N = 4 per arm) in the Phase I study and included in the PD analysis set (Table 1). All the subjects completed the study and no major protocol deviations were observed. The duration of the study was of approximately 3 months (between November 2011 and January 2012).

### Pharmacodynamics: urine pH

**Figure 3:**
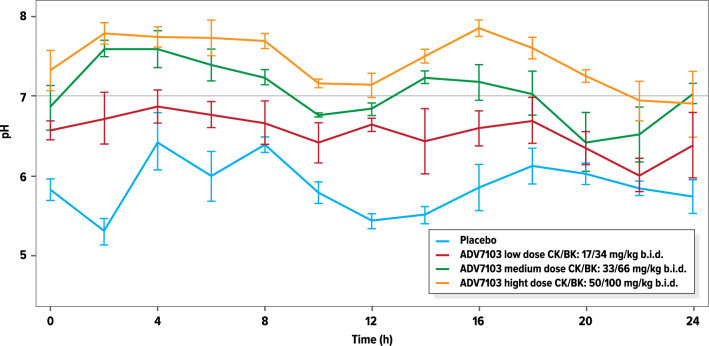
Mean (± SE) urine pH values measured at 2 h-intervals during 24 h on Day 4 of Period I (treatment administrations in the morning T = 0 h and evening T = 12 h).

Based on the mean urine pH values obtained every 2 h over a 24-h period, in the absence of treatment a strong circadian rhythm of urine pH was observed, composed of 2 cycles (Fig. 3), in agreement with the literature^11,12^. After administration of ADV7103 twice a day the circadian rhythm was still observed, with acrophase (peak) and bathyphase (trough), respectively, 2–6 h and 8–12 h after administration, but the low urine pH phases were shortened with increasing ADV7103 doses and there was an overall elevation of urine pH across all dose groups.

**Figure 4:**
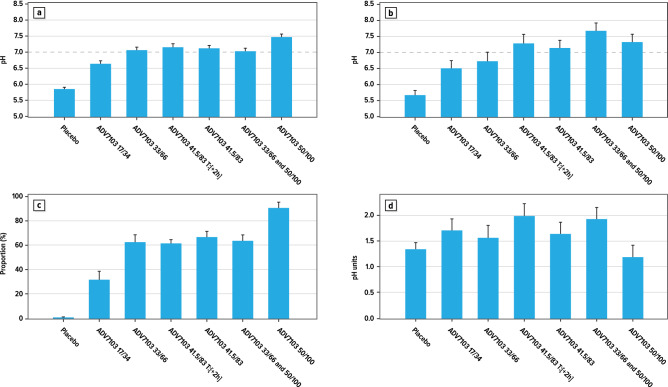
Primary endpoint measures for the different treatment doses/regimens: (**a**) Least squares mean (+ SE) 24-h urine pH (Day 4 and Day 5 pooled); (**b**) Least squares mean (+ SE) first morning and pre-dose urine pH on Day 4; (**c**) Least squares mean (+ SE) proportion of urine pH samples with pH ≥ 7 over 24 h (Day 4 and Day 5 pooled); (**d**) Least squares mean (+ SE) daily urine pH fluctuation (Day 4 and Day 5 pooled).

Treatment comparison of the different urine pH endpoints is shown in Fig. 4. The mean 24-h urine pH values were between 7.0 and 7.5 in all ADV7103 groups except for the low dose (Fig. 4a). All mean daily urine pH values were significantly increased under ADV7103 as compared to placebo (p < 0.0001 in all cases). The increase was significantly dose-related between doses in Period I. At the high dose (50/100 mg/kg CK/BK *b.i.d.*) urine pH values were statistically significantly increased compared to the intermediate (33/66 mg/kg CK/BK *b.i.d.*) and low doses (17/34 mg/kg CK/BK *b.i.d.*) (p = 0.0009 and p < 0.0001, respectively) and urine pH at the intermediate dose was statistically significantly increased compared to the low dose (p = 0.0006). There was no statistically significant difference between the different dose regimens in Period II and none of the alternative regimens seemed to present an advantage.

**Figure 5:**
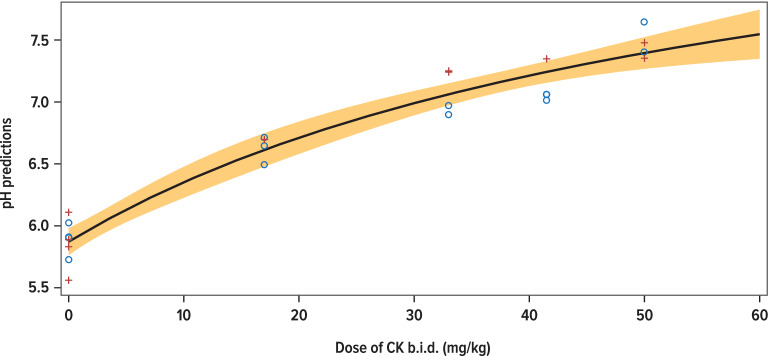
Predicted values of mean urine pH according to the dose of CK (mg/kg) in male (Ο) and female (+) subjects.

The dose-related change in mean 24-h urine pH followed an E_max_ model, as shown in Fig. 5 (ADV7103 treatments with 41.5/83.0 mg/kg CK/BK T[+ 2 h] and ADV7103 33/66 + 50/100 mg/kg CK/BK were excluded from the analysis). From that model, the dose required to reach a mean daily urine pH of 7.0 would correspond to 30.4/60.8 mg/kg CK/BK and to reach a mean daily urine pH of 7.5 a dose of 56.7/113.4 mg/kg CK/BK *b.i.d.* would be needed.

The urine pH values of the first morning pre-dose urines on Day 4 were also statistically significantly increased after ADV7103 as compared to placebo (Fig. 4b). This was demonstrated for the 3 different doses (p < 0.0001, p = 0.0015, and p = 0.0087, for the high, intermediate and low doses in Period I, respectively), and for the 3 different dosing regimens (p < 0.0001 in all cases in Period II).

The dose required to target an increase of 0.2 pH units the first morning pre-dose urine pH value on Day 4, as estimated from the corresponding E_max_ model (not shown), was 3.5/7.0 mg/kg of CK/BK *b.i.d*. For an increase of 0.5 pH units the estimated dose was 9.9/19.8 mg/kg of CK/BK *b.i.d*.

The analysis of the mean proportion of urine samples with pH ≥ 7.0 during 24 h indicated that approximately 30% of the urine pH values observed were ≥ 7.0 with the low dose (Fig. 4c). This percentage increased to about 60% of the values with the intermediate dose and the alternative regimens of Period II, and to about 90% of the values with the high dose*.* ADV7103 was shown to have alkalising power of long duration, maintaining a stable urine pH between 7.0 and 7.5 throughout 24 h, with a dosing scheme limited to two administrations a day of a dose of 50/100 mg/kg CK/BK (i.e. 1.44 meq/kg) b.i.d.

The daily fluctuation of urine pH (difference between maximal and minimal values observed over 24 h) was significantly increased after two ADV7103 regimens of Period II vs. placebo (ADV7103 41.5/83.0 mg/kg CK/BK *b.i.d*. T:[+ 2 h]; ADV7103 33/66 and 50/100 mg/kg CK/BK) (p = 0.0214 and p = 0.0337, respectively) (Fig. 4d). There were no other differences, however the daily fluctuation of urine pH tended to decrease with the highest dose (50/100 mg/kg CK/BK *b.i.d*.) compared to lower ADV7103 doses.

**Figure 6:**
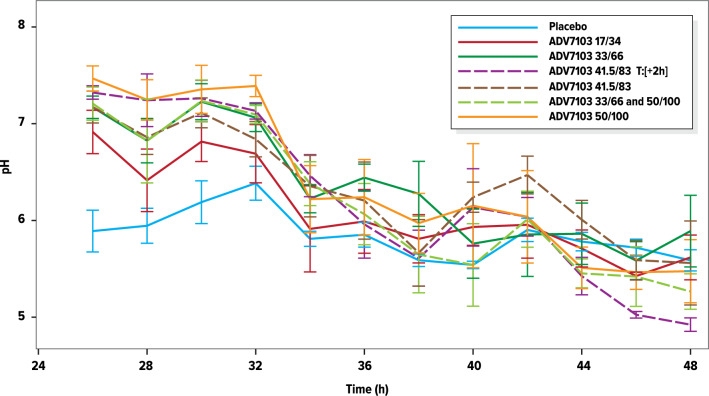
Return to baseline of urine pH values on Day 6 (both study periods).

Urine pH values returned to baseline within 24 h after the last administration of ADV7103, irrespectively of the treatment dose/regimen (see Fig. 6). In the group with the dosing regimen delayed by approximately 2 h, urine pH tended to decrease to a lower level than in the placebo group but the difference was not statistically significant (results not shown).

### Citraturia and other urine parameters

As expected, a significant and dose-dependent increase of citraturia change from baseline values was observed following oral administration of ADV7103, as compared to placebo (p < 0.05 for all doses except for the high dose for which p < 0.0001). The low dose (17/36 mg/kg CK/BK *b.i.d.*) was statistically significantly different from the high dose (50/100 mg/kg CK/BK *b.i.d.*) administered (p < 0.0447).

The comparison of urine electrolyte contents at baseline and after 4 days of treatment with the high dose of ADV7103 is shown in Table 2. Citraturia was doubled after ADV7103 treatment and calciuria was reduced, with approximately half as much urine calcium after 4 days of treatment with the high dose of ADV7103 compared to baseline. Phosphaturia was also decreased to half of the baseline values. Chloriduria and natriuria were slightly decreased but remained within normal values after ADV7103 treatment. As a consequence of the repeated potassium administration with ADV7103 treatment, kaliuria was doubled (185.8 mmol/24 h vs. 79.30 mmol/24 h at baseline).

**Table 2:** Mean ± SE urine electrolytes on 24 h-urine collections at baseline and on Day 4 after administration of ADV7103 high dose (2.88 meq/kg/day) (n = 4).

	Baseline (mmol/24 h)	After ADV7103 (mmol/24 h)
Citrate	2.45 ± 0.16	4.47 ± 0.62
Calcium	4.89 ± 1.50	2.81 ± 1.00
Chloride	176.0 ± 95.22	149.8 ± 36.72
Phosphate	40.05 ± 36.87	19.98 ± 2.90
Potassium	79.30 ± 45.81	185.8 ± 15.44
Sodium	199.3 ± 111.3	151.5 ± 37.61

### Safety

No serious adverse events or adverse events leading to treatment discontinuation occurred. A total of 31.3% (5/16) of the subjects experienced a total of 8 adverse events (AEs), all fully resolved. The proportion of patients experiencing AEs was 25% (4/16) when treated with ADV7103 and 12.5% (1/8) when receiving placebo.

Four episodes of headache of mild or moderate intensity, one episode of dizziness of moderate intensity, one mesenteric adenitis of moderate intensity, and one conjunctival irritation of mild intensity were observed, all unrelated or unlikely related to the treatment. Only one episode of nausea of mild intensity was observed 30 min after the evening administration of the high dose of ADV7103 (50/100 mg/kg CK/BK *b.i.d.*) after three days of administration. Considering to the mode of action of citrate and bicarbonate salts, the knowledge of adverse events typically related to these salts and the particular characteristics of the patients, together with the context in which the events were observed, this was the only AE considered by the investigator as possibly related to the study treatment during the whole study.

No clinically relevant findings were observed in clinical examination, laboratory parameters, vital signs or ECG parameters. Despite increased potassium administration with increasing doses of ADV7103, mean blood potassium levels were not significantly modified (mean values ≤ 4.55 mmol/L and mean change from baseline values ≤ 0.275 mmol/L for all treatment doses/regimens on Day 5). The incidence of urine pH > 8.0 was low (incidence of 0.7% (13 out of 1,750 urine collections) and without exceeding urine pH values of 8.25.

Overall, ADV7103 was well tolerated, including a good gastrointestinal tolerability, and no safety concerns were raised. The Phase I study allowed concluding that ADV7103 could be administered safely to patients requiring alkalising treatments.

## Discussion

The development of a prolonged-release oral formulation seemed the most straight-forward option to reduce the number of daily intakes while maintaining a therapeutic sustained activity of the active principles for an extended period (day and night) allowing a 24 h coverage with only 2 intakes a day. A granule formulation was preferred because multi-particulate forms have been shown to be particularly suitable for achieving prolonged-release oral formulations, with low risk of dose dumping and less inter and intra-subject variability in gastrointestinal transit time^13^. The individual prolonged-release formulations, as well as their combination have been patented, which is one of the reasons of the late publication of the present results.

One of the main drawbacks of current oral alkalising treatments is poor gastro-intestinal tolerability (nausea, vomiting, flatulence, abdominal pain, bloating and diarrhoea) due to the high amount of alkalising agent released in the stomach from the immediate-release formulations. Gastrointestinal problems may lead to treatment discontinuation in up to 48% of the cases^14–16^. In order to limit the amount of alkalising agent per intake and to provide a continuous alkalising coverage, the administration of the total daily dose is divided into multiple intakes, sometimes even during the night, which is generally detrimental for treatment acceptability. The prolonged-release granules of ADV7103, limiting the alkaline load in the stomach, resulted in a good gastro-intestinal tolerability, with only one episode of nausea of mild intensity observed with the highest dose of ADV7103. This feature, together with the limited number of daily intakes, should participate to improve compliance to long-term treatment.

ADV7103 granules are indeed two-millimetre diameter tablets manufactured by compression and film coated with a reduced amount of coating material. Compared to irregularly shaped granules, reproducibility of compression process and coating conditions are additional advantages of regularly shaped units^17^, such as those of ADV7103. The size of the granules was also considered carefully. Two-millimetre granules have shown a favourable acceptance compared to other liquid formulations even in young children^18^. This diameter size is considered to be a good compromise between technical manufacturing constraints and the size and number of granules to be swallowed^19^. Adequate coating ensures taste masking and good palatability. Indeed, potassium citrate and potassium bicarbonate are known to have a poor taste, which could also lead to compliance issues.

By virtue of the specific features of each type of prolonged-release granules and to the proportion of CK and BK, the ADV7103 formulation allows a continuous dissolution of alkalising agents and of potassium during at least 10 h.

The main limitation of this study is that it was not possible to determine the pharmacokinetic profile of the product and to evaluate its effect directly in blood, because the active substances are also endogenous compounds and in healthy subjects the homeostasis system is fully functional. Therefore, in order to evaluate the prolonged-release of ADV7103 in humans, its ability to modify urine pH over time was assessed.

Pharmacodynamic evaluations have demonstrated that ADV7103 is a strong alkalising drug with a duration of action of 24 h after two daily administrations at the appropriate dose. As expected, increasing the dose increased urine pH values proportionally, this effect being directly linked to the homeostatic effect of regulation of blood pH; No saturating effect was observed at the tested doses.

Existing alkalising agents are able to increase the urinary pH when compared to the corresponding placebo values but their duration of action is limited, requiring several daily administrations. The only alkalising therapy for which an extended release is claimed consists of a potassium citrate formulation (UROCIT-K tablets) but it has, however, been shown to exhibit a comparable profile to an immediate release liquid formulation of potassium citrate^20^. The period for which the urine pH was above 7.0 was short-lived and similar for both the liquid and the tablet formulation tested at the same dose (pH > 7.0 for only 3–4 h, approaching values observed in the placebo groups shortly thereafter). This could be explained by the limitations due to the absorption of citrate only in the first part of the intestine. In contrast, at approximately equivalent oral doses of alkalising agent, a sustained effect is observed with ADV7103 (i.e. with the medium dose of 33/66 mg/kg CK/BK), reaching urine pH values above 7.0, which confirms the advantage of ADV7103 pronged-release formulation over currently available formulations.

After 5 days of treatment with ADV7103, the composition of urine in healthy adult subjects was also modified. With the high dose, a twofold increase of citraturia was observed, while calciuria and phosphaturia were reduced to a half of their baseline values and natriuresis was also slightly reduced. The decrease of calciuria could be related to the well-known calcium chelating effect of citrate^21,22^. The short-term evolution of citraturia and calciuria under ADV7103 treatment would be in favour of a reduced risk of nephrocalcinosis or nephrolithiasis at the long-term in patients presenting elevated calcium urine levels^23–25^. As expected, in response to the potassium load provided by ADV7103, potassium levels were increased in urine. The decrease of sodium in urine could be a consequence of the increased kaliuria, due to the inverse exchange of cations in the renal tubule^26^.

At the tested doses in healthy adult subjects, the prolonged-release formulation of ADV7103 allows providing alkalising treatment with a good safety profile even for the highest doses tested (good gastro-intestinal tolerability, urine pH generally below 8.0).

By virtue of the prolonged alkalising effect of the formulation, its expected effect on preventing nephrolithiasis and its good gastrointestinal tolerability, ADV7103 could be used as an alternative to current treatments in different indications such as distal renal tubular acidosis and cystinuria but further evaluations are warranted.

In conclusion, the present fixed-dose combination of potassium citrate and potassium bicarbonate, formulated as prolonged-release granules, could allow a sustained alkalising effect with only two administrations a day. It could be suitable for children and adults, due to its multi-particulate form allowing dose adaptation and low risk of dose dumping. Results of increased urine pH maintenance together with an overall good safety, including very good gastrointestinal tolerability in healthy subjects, support further clinical evaluation of ADV7103 in patients requiring treatment with alkalising agents. Reduced inter and intra-subject variability in gastro-intestinal transit time, limited risk of local gastro-intestinal events, good palatability and ease of administration in order to improve compliance are also expected but further clinical investigations in paediatric and adult patients are required to confirm the prolonged effect, acceptability and safety of ADV7103 formulation.

## References

[1] Pak CY, Fuller C, Sakhaee K, Preminger GM, Britton F (1985). Long-term treatment of calcium nephrolithiasis with potassium citrate. J. Urol..

[2] Jendle-Bengten C, Tiselius HG (2000). Long-term follow-up of stone formers treated with a low dose of sodium potassium citrate. Scand. J. Urol. Nephrol..

[3] Grases F, Conte A, March JG, Garcia-Ferragut L (1998). Evolution of lithogenic urinary parameters with a low dose potassium citrate treatment. Int. Urol. Nephrol..

[4] Hamm LL (1990). Renal handling of citrate. Kidney Int..

[5] Caudarella R, Vescini F, Buffa A, Stefoni S (2003). Citrate and mineral metabolism: kidney stones and bone disease. Front. Biosci..

[6] Gleeson D (1992). Acid-base transport systems in gastrointestinal epithelia. Gut.

[7] Sladen GE (1971). The problem of measurement of intestinal absorption in man. Proc. R. Soc. Med..

[8] Turnberg LA, Fordtran JS, Carter NW, Rector FC (1970). Mechanism of bicarbonate absorption and its relationship to sodium transport in the human jejunum. J. Clin. Invest..

[9] Fjellstedt E, Denneberg T, Jeppsson JO, Tiselius HG (2001). A comparison of the effects of potassium citrate and sodium bicarbonate in the alkalinization of urine in homozygous cystinuria. Urol. Res..

[10] Tekin A, Tekgul S, Atsu N, Sahin A, Bakkaloglu M (2001). Cystine calculi in children: the results of a metabolic evaluation and response to medical therapy. J. Urol..

[11] Firsov D, Bonny O (2010). Circadian regulation of renal function. Kidney Int..

[12] Cameron M (2012). The diurnal variation in urine acidification differs between normal individuals and uric acid stone formers. Kidney Int..

[13] Dey N, Majumdar S, Rao M (2008). Multiparticulate drug delivery systems for controlled release. Trop. J. Pharm. Res..

[14] Barcelo P, Wuhl O, Servitge E, Rousaud A, Pak CY (1993). Randomized double-blind study of potassium citrate in idiopathic hypocitraturic calcium nephrolithiasis. J. Urol..

[15] Ettinger B (1997). Potassium-magnesium citrate is an effective prophylaxis against recurrent calcium oxalate nephrolithiasis. J. Urol..

[16] Hofbauer J, Hobarth K, Szabo N, Marberger M (1994). Alkali citrate prophylaxis in idiopathic recurrent calcium oxalate urolithiasis: a prospective randomized study. Br. J. Urol..

[17] Tissen C, Woertz K, Breitkreutz J, Kleinebudde P (2011). Development of mini-tablets with 1 mm and 2 mm diameter. Int. J. Pharm..

[18] Klingmann V (2013). Favorable acceptance of mini-tablets compared with syrup: a randomized controlled trial in infants and preschool children. J. Pediatr..

[19] van Riet-Nales DA (2015). Methods of administering oral formulations and child acceptability. Int. J. Pharm..

[20] Harvey JA, Zobitz MM, Pak CY (1989). Bioavailability of citrate from two different preparations of potassium citrate. J. Clin. Pharmacol..

[21] Coe FL, Favus MJ (1992). Disorders of Bone and Mineral Metabolism.

[22] Tiselius HG, Fornander AM, Nilsson MA (1993). The effects of citrate and urine on calcium oxalate crystal aggregation. Urol. Res..

[23] Pak CY, Sakhaee K, Fuller C (1986). Successful management of uric acid nephrolithiasis with potassium citrate. Kidney Int..

[24] Preminger GM, Sakhaee K, Skurla C, Pak CY (1985). Prevention of recurrent calcium stone formation with potassium citrate therapy in patients with distal renal tubular acidosis. J. Urol..

[25] Suarez M, Youssef F (2015). Potassium citrate: treatment and prevention of recurrent calcium nephrolithiasis. J. Clin. Nephrol. Res..

[26] Palmer BF (2015). Regulation of potassium homeostasis. Clin. J. Am. Soc. Nephrol..

